# Do Breast Cancer Cell Lines Provide a Relevant Model of the Patient Tumor Methylome?

**DOI:** 10.1371/journal.pone.0105545

**Published:** 2014-08-26

**Authors:** Leslie M. Cope, Mary Jo Fackler, Zoila Lopez-Bujanda, Antonio C. Wolff, Kala Visvanathan, Joe W. Gray, Saraswati Sukumar, Christopher B. Umbricht

**Affiliations:** 1 Department of Oncology, The Johns Hopkins University School of Medicine, Baltimore, Maryland, United States of America; 2 Department of Surgery, The Johns Hopkins University School of Medicine, Baltimore, Maryland, United States of America; 3 Department of Pathology, The Johns Hopkins University School of Medicine, Baltimore, Maryland, United States of America; 4 Department of Epidemiology, Bloomberg School of Public Health, Baltimore, Maryland, United States of America; 5 Department of Biomedical Engineering, Oregon Health & Science University, Portland, Oregon, United States of America; Rutgers - New Jersey Medical School, United States of America

## Abstract

It is well documented that tumor cells undergo dramatic genetic and epigenetic changes during initial establishment as cell lines and in subsequent serial passaging, and that the resultant cell lines may have evolved significantly from the primary tumors from which they were derived. This has potential implications due to their widespread use in drug response experiments and studies of genomic function. One approach to optimizing the design of such cell line studies is to identify and use the cell lines that faithfully recapitulate critical features of primary tumors. To evaluate the epigenetic fidelity of breast cancer cell lines in the context of primary tumors, we performed methylation profiling of 55 well-characterized breast cancer cell lines on the Illumina HumanMethylation27 BeadChip platform, and compared them to publicly available methylation profiles of primary breast tumors. We found that the DNA methylation profiles of breast cancer cell lines largely retain the features that characterize primary tumors, although there are crucial differences as well. We describe these similarities and differences between primary tumors and breast cancer cell lines in detail, and develop a quantitative measure of similarity that is used to score each cell line with respect to how faithfully its methylation profile mirrors that of primary tumors.

## Introduction

The statistician George Box might have been thinking of cell line models of human cancer when he said, “All models are wrong; some models are useful.” On the one hand, studies on drug response and genomic function which are often performed using cell lines are carried out with comparable ease and speed compared to more complex, in vivo model systems. On the other hand, tumor cells may undergo dramatic genetic and epigenetic changes during establishment in culture as cell lines, and continue to do so in serial passages, potentially resulting in cell lines that show limited resemblance to the primary tumors from which they were derived [1]–[3].

The success of recent studies using the COXEN approach [4] to translate markers of drug response from cell lines to primary tumors [5]–[8] highlights both the risks and the rewards of using cell line models of cancer. These studies successfully stratified responders and non-responders to several drugs in a variety of tumor types using marker signatures derived from experiments on cell lines. The success of this strategy could be attributed to the fact that the candidate markers had been carefully selected to determine which ones had expression concordant to that of primary tumors [4]. Thus, understanding the ways in which cell lines are similar to primary tumors is a prerequisite to the optimal design of pre-clinical cell line studies. This knowledge makes it possible to determine which genes faithfully recapitulate the most important features of primary tumors, while avoiding cell lines that appear to have evolved significantly and have diverged in some critical features relevant to a particular study.

Several studies have documented similarities and differences between breast cancer cell lines and primary tumors at the level of gene expression [9], [10], DNA copy number [9] and in response to therapy [11]. This is the first comprehensive comparison of breast cell lines and primary tumors using DNA methylation profiles.

Tumor suppressor gene expression is frequently transcriptionally down-regulated by DNA hyper-methylation of the gene promoter region [12], [13]. DNA methylation changes are stable and inheritable, but unlike mutations and copy number alterations in the genetic code, they are potentially reversible [14], [15]. Epigenetic characterization of tumor cell lines could contribute to their use as models of in vivo processes such as drug response, and the comparison of their methylation signatures with those of patient tumors can provide guidance for their appropriate selection and use. Since the tumors of origin are no longer available in most cases, we have used publicly available data on breast cancer DNA methylation profiles as reference for this study.

A number of publications recently reported methylation profiles of primary breast tumors, including Dedeurwaerder et al. [16], Fackler et al. [17], Fang et al. [18], and the collaborative effort reported by the TCGA consortium [19]. In addition to DNA methylation profiles, all four studies reported immunohistochemical markers (ER, PR and HER2). Gene expression measurements were also reported for all TCGA samples and for a subset of the samples from the Dedeurwaerder study [16]. Although each of these studies has a specific focus and reports unique results, there was also a clear consensus on several key findings. All four of these studies observed distinct methylation profiles associated with ER status, and noted that ER+ tumors generally have higher overall levels of promoter DNA methylation than ER- tumors. Additionally, Fang and TCGA both reported distinct breast cancer CpG-island hyper-methylator or B-CIMP phenotypes [18], [19], and both Dedeurwaerder and TCGA reported distinctive tumor subtypes based on methylation profiles [16], [19]. Moreover, Fang found that among ER+ tumors, higher global levels of promoter DNA methylation are associated with less aggressive disease [18].

In the present study, we have undertaken the first systematic, genome-wide comparison of DNA methylation profiles of cancer cell lines and primary tumors using a panel of 55 well-characterized breast cancer cell lines [9]–[11], and publicly available primary tumor datasets [16]–[19].

We describe similarities and differences between primary tumors and breast cancer cell lines in detail, focusing on the methylation-based subtypes, CpG island methylator phenotype and correlations to ER status that have been reported in primary tumor studies. We report the development of a quantitative measure of similarity that we used to score each cell line with respect to how faithfully its methylation profile mirrors that of primary tumors.

## Materials and Methods

### Cell lines

The 55 breast cancer cell lines used in this study (Table S1) were collected by Dr. Joe Gray from the ATCC or from collections developed in the laboratories of Drs. Steve Ethier and Adi Gazdar as previously described [9], [10] and provided by the NCI (IBC45) through a contract with ATCC to our laboratory.

### Methylation array analysis and validation

DNA was extracted as previously described [9]. Sodium bisulfite-conversion was performed using the EZ DNA methylation Kit (Zymo Research, D5002), column-purified using a ZymoSpin IC column, and eluted in 12 µl of water. The eluted DNA was quantified and hybridized to Illumina Infinium Human Methylation27 BeadChip Kit (WG-311-1202) in the DNA Microarray Core at the Sidney Kimmel Cancer Center at JHU. To minimize technical artifacts, samples were arrayed as a single batch.

### Array Data analysis

Data were extracted using GenomeStudio Methylation Module v1.0 software. The methylation value for each 50 bp CpG locus is expressed as a β-value, representing a continuous measurement from 0 (completely unmethylated) to 1 (completely methylated) according to the following calculation: β value  =  (signal intensity of M probe)/(signal intensity of M+U probes). Full methodological details for performing the methylation array analysis are provided in our earlier publication [17]. All cell line methylation data have been deposited in the Gene Expression Omnibus (http://www.ncbi.nlm.nih.gov/geo/), with the accession number GSE42944.

### Quality Control of array findings

The methylation levels of several genes were evaluated in the same cell lines using an independent assay, Quantitative Multiplex Methylation Specific PCR (QM-MSP) [17], [20]. In addition, the published data on DNA methylation profiles of the TD47 and MCF7 cell lines [21], independently verified using a sequence-based method, Methyl-MAPS [22], was downloaded from the Gene Expression Omnibus (GSE45337) to further establish the quality and accuracy of our array results.

In addition to the methylation profiles prepared for this study, copy number and gene expression profiles were obtained for 50 of the breast cancer cell lines using the Affymetrix GeneChip Human Genome U133A (hgU133A) array [9], [10], and an extensive drug response study by the Gray lab incorporated most of these cell lines as well [11]. ER, PR and HER2 status for the cell lines were characterized by immunohistochemistry (and by FISH in the case of HER2), and for those included in the expression analyses, the expression-based subtypes, designated *luminal, basal A* and *basal B*, were assigned by clustering sample profiles using the most variable expression probes as described previously [9], [10]. We combined the two sources of IHC and expression-based subtype data, primarily following the characterization by Kao et al. [10], but using data presented in the earlier study by Neve et al. [9] for cell lines not described in Kao.

Several additional publicly available datasets were used in the course of analysis, including DNA methylation profiles of primary breast cancer tumors by Fackler et al. [17], Fang et al. [18], Dedeurwaerder et al. [16], and the Cancer Genome Atlas Project (http://cancergenome.nih.gov/). Additional publicly available expression datasets used in this investigation included studies by Farmer et al. [23], and Sotiriou et al. [24].

### Analytic methods

All analyses were performed in R (***cran*** *.us.r-project.org/*) using both published packages and custom routines. Heatmaps were generated using the **pHeatmap** package (http://cran.r-project.org/web/packages/pheatmap/index.html). Unsupervised agglomerative hierarchical clustering [25] was performed with Ward's minimum variance method [26], based on methylation patterns in the most variable 2% of CpG sites. We developed a global methylation score to summarize total promoter, DNA methylation in each sample, across the highly variable sites used for clustering. The data for each probe was first standardized to have a mean of zero and standard deviation of one, and then standardized values were averaged across probes, within sample, to generate a sample specific score. A value above zero indicates that a sample is more frequently methylated than average while a value below zero indicates lower than average methylation. ANOVA was performed in R, and summarized using F-tests with Type I error controlled at α = 0.05.

### Defining signatures of methylation phenotypes

Published methylation signatures derived on primary tumors were used to compare cell lines to primary tumors, including the 200 probe signature of predicted ER status defined by Fackler et al. [17] and the taxonomy of breast cancer methylation subtypes described by Dedeurwaerder et al. [16]. For the definition of B-CIMP from Fang et al. [18], where 3297 probes were found to be correlated with B-CIMP, we selected a subset of 1440 probes that had a false discovery rate below 0.01.

### Predicting phenotype by translating gene signatures from primary tumors

A 3-step process was used to map phenotypes identified in one of the primary tumor studies to cell lines and additional primary tumor datasets: 1) The methylation levels at each locus were standardized within the dataset by subtracting the mean and dividing by the standard deviation. 2) The mean, within-group methylation level for each probe was calculated in the training dataset for each phenotypic group, to define a typical methylation profile for the class. 3) Individual cell lines and samples from independent test datasets were correlated to the class-specific prototypes by Spearman correlation, and assigned to the best correlated class.

### Matching individual cell lines to individual primary tumors by nearest neighbor analysis

Using all probes on each array, the Spearman Correlation Distance was calculated between each pair of samples to identify the best primary tumor match for each cell line using the **bioDist package** (http://www.bioconductor.org/packages/release/bioc/html/bioDist.html). The distance to the nearest tumor was used to measure how closely each cell line resembled primary tumors.

## Results

### Genome-wide methylation profiling of breast cancer cell lines

**Figure 1:**
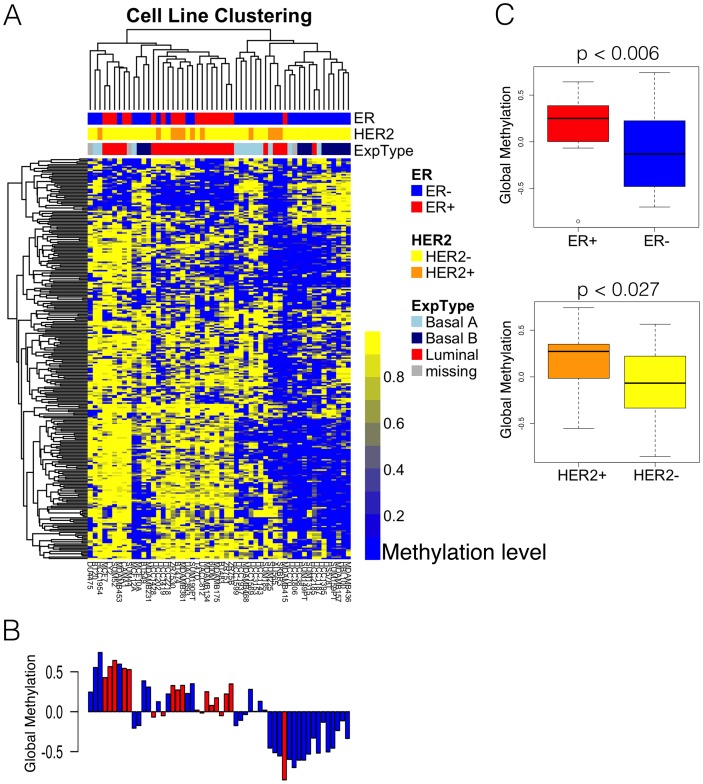
Genome-wide methylation patterns in breast cancer cell lines. Panel A shows a heat map of DNA methylation resulting from an unsupervised cluster analysis of highly variable loci (defined as standard deviation in top 1%). The methylation level for each 50 bp CpG locus is expressed as a β-value ranging from 0 (blue) to 1 (yellow), calculated as described in the methods section. Gene expression-based categories of Luminal, Basal A, and Basal B as defined by Neve et al. [9] are annotated in color along the top (Luminal in red, Basal A in light blue and Basal B in dark blue), as are the immunohistochemically defined ER (ER+ in red, ER- in blue) and HER2 status (HER2+ in orange, HER2- in yellow) of each cell line. In Panel B, our *DNA hypermethylation score*, summarizing methylation levels across the genome (see Methods for details), depicts standardized average methylation levels for samples represented in same order as panel A. A value above zero indicates that a sample is more frequently methylated than average, while a value below zero indicates lower than average methylation. ER status is indicated by the color of the bar (ER+ in red, ER- in blue). The boxplots in Panel C summarize the distribution of hypermethylation scores according to ER status above (t = 2.93, p-value = 0.0055), and HER2 status below (t = 2.31, p-value = 0.027), again using the standardized average methylation levels.

In order to characterize the overall distribution of DNA methylation in breast cancer cell lines, we performed an unsupervised cluster analysis of DNA methylation levels on the 2% of CpG loci with the largest standard deviation across cell lines. The resulting heatmap, shown in Figure 1A, reveals that patterns of DNA methylation broadly segregate breast cancer cell lines according to ER status, and more specifically into the expression-based categories of Luminal, Basal A, and Basal B previously described [9]–[11]. In fact, in these cell lines, methylation levels at thousands of loci are significantly associated with the expression-based subtypes. Interestingly, the sharpest distinction can be drawn between Basal B cell lines and all others (Basal A and Luminal samples combined). Full probe-by-probe results are provided in Table S1.

The summary scores of overall DNA methylation levels in these expression subtype-associated loci are shown in Figure 1B and 1C, and differ dramatically (p-value = 0.0055) between ER+ and ER- lines, with ER+ showing substantially higher levels of methylation. HER2 overexpression is less strongly correlated with higher methylation levels, an association that may be wholly driven by associations between ER and HER2 status (p-value = 0.027). These findings confirm results from recent studies of primary breast tumors [16]–[18], all of which found that the well-known breast cancer subtypes have distinctive methylation patterns involving hundreds or even thousands of genes, and that luminal tumors have overall higher levels of methylation. The initial conclusion from these results is that tumor-specific DNA methylation changes are likely to play key roles in the biological processes that characterize breast tumors.

To verify our array data using an independent assay, we determined the methylation levels of several genes previously known to be methylated in primary tumors by QM-MSP [17]. In all four cases, the assay results were highly concordant, with correlation coefficients ranging between 0.85 and 0.98, as shown in Figure S1. Additionally we downloaded sequence-based Methyl-MAPS methylation profiles for two of the cell lines, MCF7 and TD47, and compared results across the genome using array probes within 100 base pairs of a Methyl-MAPS measurement. Here, again, we observed good fidelity between platforms as shown in Figure S2.

### Cell line methylation profiles are similar, but not identical to primary breast cancers

Next, we sought to determine if specific results on genome wide methylation reported for primary breast cancer cohorts are also reflected in the cell line models. While correlations between methylation and outcome cannot be tested in cell lines, other findings, identified in several primary tumor studies and validated in independent samples, can be evaluated. These include: 1) the presence of a CpG island methylator phenotype (CIMP) identified in a subset of ER+ tumors [18]; 2) a taxonomy of 6 breast tumor subtypes defined by characteristic methylation profiles [16]; and 3) a methylation signature strongly associated with ER status [17]. Using the correlation based procedures detailed in Methods, each cell line was matched to one of the 6 Dedeurwaerder subtypes, characterized as to CIMP status, and assigned to a predictive ER class, according to the its methylation pattern in the respective signature. To provide an independent comparison between cell lines and primary tumors, we identified two additional publicly available sets of breast tumors analyzed on the Illumina HM27 methylation platform, inferring phenotypes for these samples as well. One of these, herein called *Dedeurwaerder Validation*, was previously used as an independent validation set for the Dedeurwaerder methylation subtypes [16]. The other, from the Cancer Genome Atlas Project (http://cancergenome.nih.gov/), and referred to here as *TCGA*, was used by both Fackler and Fang for validation [17], [18], but neither were used for signature development.

**Figure 2:**
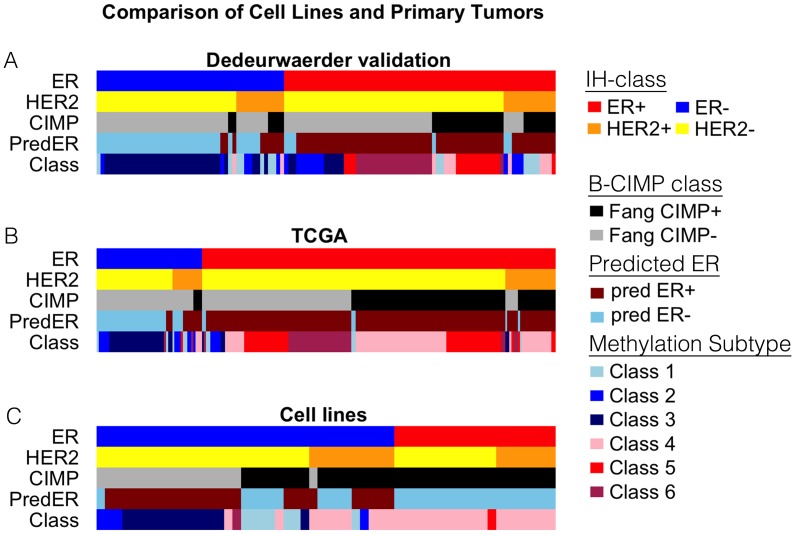
Some triple-negative cell lines have luminal-like methylation features. Composite color maps of phenotypic features of two primary breast tumor sets that were *not* used to derived any of the predictive gene signatures, i.e., the Dedeurwaerder validation set (A), and the TCGA tumor set (B), as well as our collection of breast cell lines (C) are shown. In each of the three phenotypic maps A-C, standard immunohistochemical parameters (IH class) are shown in the ER and HER2 rows. The three additional rows show B-CIMP status as predicted by Fang et al. (CIMP) [18], Methylation-based predicted ER status (Pred ER) [17], and Dedeurwaerder methylation subtype (Class) [16], all of which were predicted on the basis of gene methylation signatures as described in Methods.

The prototypical tumor-derived methylation phenotypes are shown in the supplementary figures for B-CIMP (Figure S3A), predicted ER status (Figure S4A), and Dedeurwaerder methylation subtypes (Figure S5A), respectively. Profiles for cell lines are shown in Panels S3B, S4B, and S5B, while TCGA samples and Dedeurwaerder validation samples are shown in Panels C and D of Figures S3-S5. For each signature, the distribution of methylation levels in TCGA and Dedeurwaerder validation samples (Panels C and D) is similar to that of the respective primary tumor discovery set shown in each respective Panel A, while the cell lines (Panels B) exhibit significant departures from the common primary tumor patterns. The resulting class predictions are shown in Figure 2 and itemized in Table S2.

In Figure 2, values of ER and HER2 measured by immunohistochemistry (IHC) and fluorescent in-situ hybridization (FISH) are shown alongside the 3 phenotypic variables (B-CIMP, Methylation-Predicted ER, Methylation class) that were inferred as described above. One immediately evident feature of the cell line profiles is the absence of an ER+/CIMP- phenotype, a profile that can be seen prominently in both primary tumor sets.

A second distinctive feature of the cell line profile is that nearly 20% of the cell lines are ER-, but show the B-CIMP methylation pattern, a combination that is nearly absent in primary tumor profiles.

### Methylation profiles of luminal-like triple-negative cell lines show least resemblance to primary tumors

To this point, analysis has been focused on subtype analyses of the tumors and cell lines. To focus on individual cell lines, we used a nearest neighbor analysis, described in the Methods section, to calculate pairwise distances between individual cell lines and individual primary tumors. This enabled us to associate each cell line to a nearest-matching primary tumor by both methylation and expression profiles, and thereby identify cell lines that are particularly poor matches to any primary tumor.

**Figure 3:**
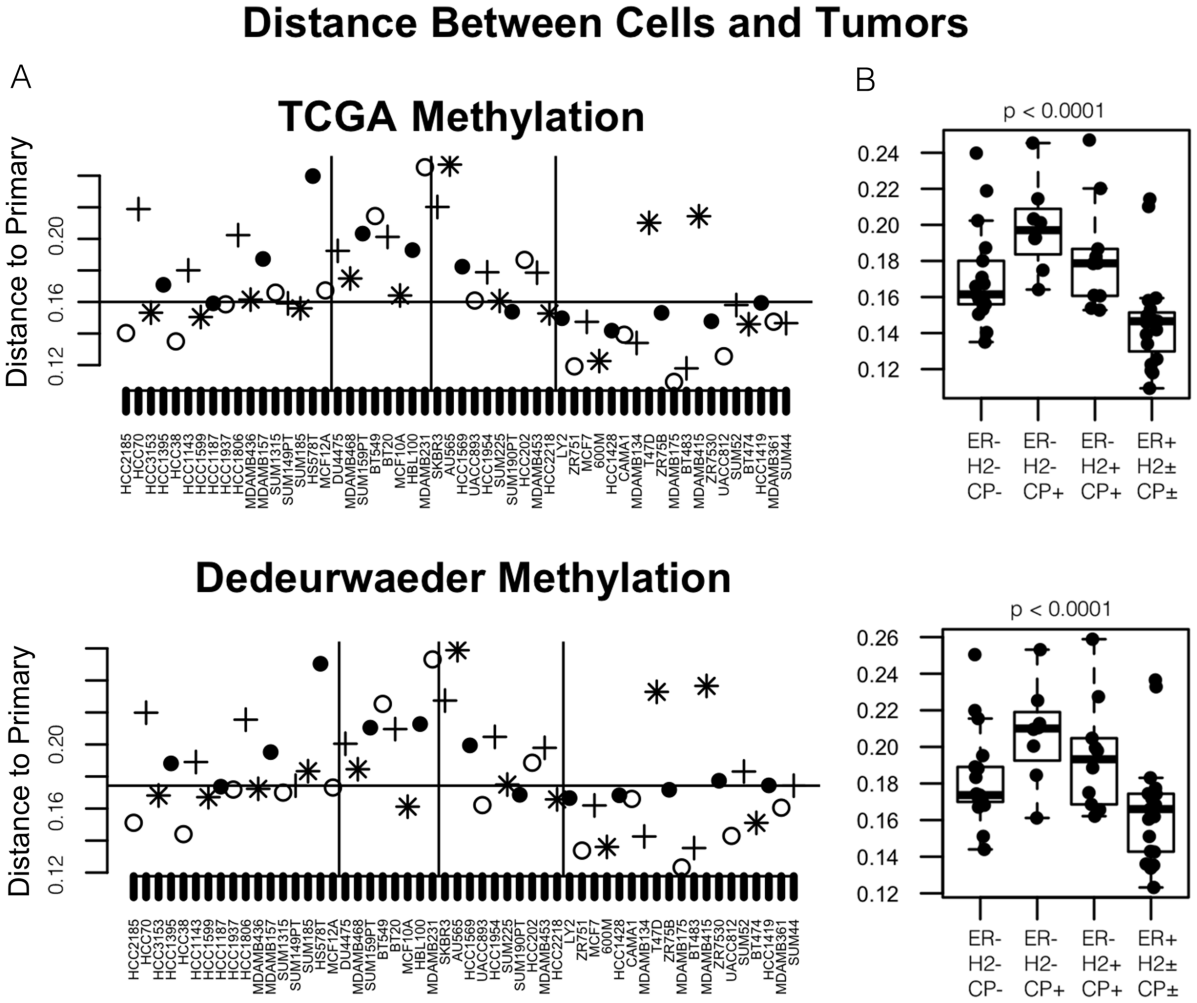
Methylation profiles of B-CIMP+, luminal-like triple-negative cell lines show least semblance to primary tumors. Panel A: The x-axis of each plot indexes cell lines, which are arranged into the following four phenotypic groups, separated by vertical bars, from left to right: 1) ER-/HER2-/B-CIMP-; 2) ER-/HER2-/B-CIMP+; 3) ER-/HER2+/B-CIMP+; and 4) ER+. ER and Her2 status are defined by IHC, and B-CIMP status by methylation profile. The y-axis shows the Spearman Correlation Distance (“Distance to Primary”) to the nearest primary tumor by methylation profile (see methods for the details of the distance measure). In the top panel, the comparison is made to TCGA HM27 samples, while in the bottom panel, the comparison is to the Dedeurwaerder validation dataset. The median distance for all 55 cell lines is shown as a horizontal line as a reference point, and symbols of adjacent points alternate to aid in tracking results across the two plots. Panel B: The boxplots show the distribution of best-match distances, broken down by the major phenotypic combinations indicated by vertical bars in Panel A. Abbreviations: ER: Estrogen receptor; H2: Her2 amplification; CP: B-CIMP status.

For this purpose, we again used the TCGA (http://cancergenome.nih.gov/) and Dedeurwaerder Validation [16] DNA methylation studies, as well as two public primary tumor expression datasets based on hgU133A arrays [23], [24]. The results are shown in Figure 3 for nearest neighbors calculated using all probes on the methylation array. We considered several subsets of probes as well, but outcomes were nearly identical (data not shown). As the boxplots in Figure 3b show, ER+ cell lines tend to have closer primary tumor neighbors than do ER- samples. The anomalous B-CIMP+, ER- breast cancer cell lines are distinctive as a group, being least likely to have a close neighbor among primary tumors. This was true irrespective of HER2 status. Analysis of Variance to compare distance to primary tumor by group was statistically significant with p<0.0001.

### Gene expression profiles of luminal-like triple negative cell lines are least like primary tumors as well

**Figure 4:**
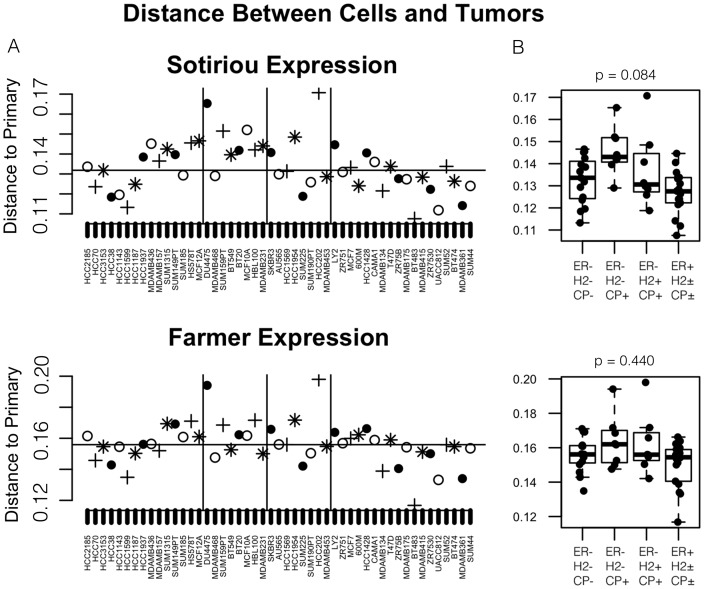
Gene expression profiles of luminal-like triple negative cell lines are least like primary tumors as well. Comparison of each individual cell line expression profiles to their closest primary tumor match supports the finding from our analysis of methylation profiles that B-CIMP+, triple-negative breast cancer cell lines are among the least likely to have a close match. Panel A: The x-axis of each plot indexes cell lines, arranged by phenotypic status, and the y-axis shows the Spearman Correlation Distance to the nearest primary tumor by expression profile. In the top half of the figure, the comparison is made to Sotiriou samples [23], [24], while in the bottom half, the comparison is made to the Farmer validation dataset [23], [24]. The median distance for all 50 cell lines is shown as a horizontal line as a reference point, and the color of adjacent points alternates to aid in tracking results across the two plots. Panel B: Boxplots in show the distribution of best-match distances, broken down by major phenotypic combinations as in Figure 3.

Using expression data available on Affymetrix hgU133A GeneChips for 50 of the cell lines in the publicly available Breast Cancer expression data sets by Sotiriou and Farmer [24], we calculated nearest-neighbor tumors for each cell line as well, with very similar results (Figure 4). As was observed for methylation profiles, cell lines that were ER+ by expression tended to be more similar to primary tumors than ER- samples. The results mirrored those seen in the methylation data with the B-CIMP+, ER- breast cancer cell lines least likely to have a near neighbor in primary tumors, although the ANOVA test did not achieve statistical significance for the expression data.

## Discussion

This is the first comprehensive comparison of the genome-wide DNA methylation profiles of breast cancer cell lines with primary tumors. It is unique not only for its size, including 55 cell lines, which are compared to methylation profiles from 561 primary tumors from 4 published studies, but also for the depth of the comparison to primary tumors. We comprehensively evaluated DNA methylation profiles of breast cancer cell lines in comparison to primary tumors to assess the extent to which the cell line model represents tumor DNA methylation patterns.

A careful analysis of this question is critical to the design and interpretation of any study exploring drug response experiments and studies of genomic function in cancer cell lines. Tumor cells undergo significant genetic and epigenetic changes during establishment, and in later serial passages that may span decades in some instances, so that commonly used cell lines may bear little resemblance to the primary tumors from which they were derived. There is, nevertheless, no doubt that cell lines have provided valuable screening tools for drug discovery as well as the study of molecular mechanisms underlying breast cancer since the time they were first established as tumor models. As described in this paper, we find that, with some noteworthy exceptions, the DNA methylation profiles of breast cancer cell lines largely retain the features that characterize primary tumors. We have developed a quantitative measure of similarity that we use to score each cell line with respect to how faithfully its methylation profile mirrors those of primary tumors. This process led to the identification of a distinctive set of ER- breast cancer cell lines that share an unusual set of luminal-like molecular features that are rarely, if ever, found in primary tumors. This finding is, however, consistent with the hypothesis that the B-CIMP phenotype overlaps an in vitro adaptation signature. The same set of cells frequently fall into Dedeurwaerder subtype 4, which is strongly associated with ER+ phenotype in primary tumors, and most of these cell lines are also predicted to be ER+ using the Fackler methylation signature of ER status, albeit not by IHC. These luminal-like, B-CIMP positive ER- cell lines represent an intriguing class that appear over-represented in cell line collections. More generally, in primary tumors, a large proportion of ER- samples show no systematic methylation in B-CIMP-associated loci, whereas every ER- cell line shows moderate to high methylation at many of these probes, although only about half were classified as B-CIMP-positive. Significantly, in spite of its poor match to primary tumors, this ER-/CIMP+ group contains some of the best known and widely used breast cancer cell lines, including MDA-MB-468, BT20, and MDA-MB-231. For some of these cell lines, it is possible that extensive passaging has contributed to the distinctive methylation profiles seen. In some cases, a cell line may be uniquely useful in research because unusual molecular characteristics render it highly responsive to perturbation, but in the absence of tumors showing comparable characteristics, due diligence would require consideration of additional tumor models to ensure that any conclusions could apply clinically.

Similarly, an additional noteworthy finding of this analysis is the complete lack of ER+/CIMP- cell lines, although this phenotype is well represented in tumor samples; accompanied by a reciprocal lack of a tumor phenotype corresponding to the above mentioned ER-/CIMP+ cell line phenotype. Notably, in the primary tumor datasets, the ER+/CIMP- phenotype is associated with Dedeurwaerder classes 5 and 6, which largely correspond to luminal expression subtypes, and which are both very rare in the large collection of cell lines examined in this study. This observation, seen in cell lines derived largely from metastatic tumors, is in sharp contrast to Fang's finding that the B-CIMP phenotype in ER+ tumors decreases the risk for metastasis. Our results suggest that luminal type tumor cells, with generally less prominent cell proliferation markers and higher overall methylation levels, may adapt to life in vitro by expressing or re-expressing B-CIMP markers. These findings support the view that the B-CIMP phenotype may represent or overlap an in vitro adaptation signature.

One would expect similar discrepancies between tumor tissue and established cancer cell lines in additional genomic assessments, such as non-transcribed RNA profiles or DNA copy number analyses, underlining the importance of verifying the clinical relevance of cell-line based investigative models against representative tissue data such as that provided by the TCGA.

In conclusion, the fact that methylation profiles of breast cancer cell lines largely retain the features that characterize primary tumors, a finding that is encouraging for cell-line based research models. This is particularly notable for the methylome, since it is subject to environmental and other influences than can modify DNA methylation states and might be expected to be less stable than structural evolution at the DNA sequence level.

## Supporting Information

Figure S1 **A–D: Validation of array measurements by QM-MSP.** Breast cancer cell line methylation levels of selected genes measured by array (x-axis, with β-values ranging from 0–1) and independently by QM-MSP (y-axis, 0–100% methylation). Agreement between assay modalities is measured by Pearson correlation coefficient r. Results for the genes DAB2IP, EVI1, GAS7 and FZD10 are shown in panels A–D, respectively.
(TIF)

Figure S2 **A–B: Validation of array measurements by Methyl-MAPS** [22] **.** Methylation levels of the breast cancer cell lines MCF7 (Panel A) and T47D (Panel B) measured by array (x-axis, with β-values ranging from 0–1) and independently by Methyl-MAPS (y-axis, with β-values ranging from 0–1). CpG sites on the array were selected to be within 100 bp of the corresponding Methyl-MAPS site. Agreement is measured by Pearson correlation coefficient r.
(TIF)

Figure S3 **A–D: B-CIMP patterns in primary tumors and cell lines.** Panel A shows DNA methylation levels for B-CIMP markers in the Fang tumor samples used to define the B-CIMP signature. ER status, (blue vs red) is indicated along the top margin, along with B-CIMP status (black vs. white). For cell lines (Panel B), the TCGA samples (Panel C), and the Dedeurwaerder validation study (Panel D), CIMP status is inferred as described in Methods and rows in all panels are arranged to match the Fang study in Panel A rather than independently clustered. The methylation level is expressed as a β-value ranging from 0 (no methylation, blue) to 1 (complete methylation, yellow), calculated as described in the Methods section.
(TIF)

Figure S4 **A–D: ER Methylation-signature markers in primary tumors and cell lines.** Panel A shows DNA methylation levels for ER markers in the Fackler tumor samples used to define the ER methylation signature [17]. For each tumor, ER status (blue vs red) and HER2 status (yellow vs orange) as defined by immunohistochemistry are indicated along the top margin, along with the ER status predicted by the ER methylation signature as described in methods. For cell lines (Panel B), the TCGA samples (Panel C), and the Dedeurwaerder validation study (Panel D), IHC-defined ER and Her2 status as well as predicted ER status are shown and rows are arranged to match the Fackler study in Panel A rather than independently clustered.
(TIF)

Figure S5 **A–D: Markers of methylation-based subtypes in primary tumors and cell lines.** Panel A shows DNA methylation levels for markers used to define the signature of the methylation-based subtypes in the Dedeurwaerder tumor samples. ER status as defined by IHC, as well as methylation class, are annotated along the top margin for each tumor sample. For cell lines (Panel B), the TCGA samples (Panel C), and the Dedeurwaerder validation study (Panel D), Dedeurwaerder classes are inferred from the markers as described in methods and rows are arranged to match the Dedeurwaerder study in Panel A rather than independently clustered.
(TIF)

Table S1 **Molecular characteristics of cell lines.** ER, PR and HER2 status for each cell line, as measured by IHC and FISH, expression and methylation subtypes, predicted B-CIMP status and predicted ER status using the Fackler methylation markers are recorded for each cell line.
(XLS)

Table S2 **DNA methylation by expression subtype.** Results from ANOVA analysis evaluating probe-specific methylation by expression subtype are shown, including the F-statistic, p-value and Benjamini-Hochberg adjusted p-value. The mean beta value for each expression group is shown as well.
(XLS)
