# Supplementary figures and images for: Do Breast Cancer Cell Lines Provide a Relevant Model of the Patient Tumor Methylome?
Source: PLoS One. 2014 Aug 26;9(8):e105545. doi: 10.1371/journal.pone.0105545 (PMC4144876; doi:10.1371/journal.pone.0105545)

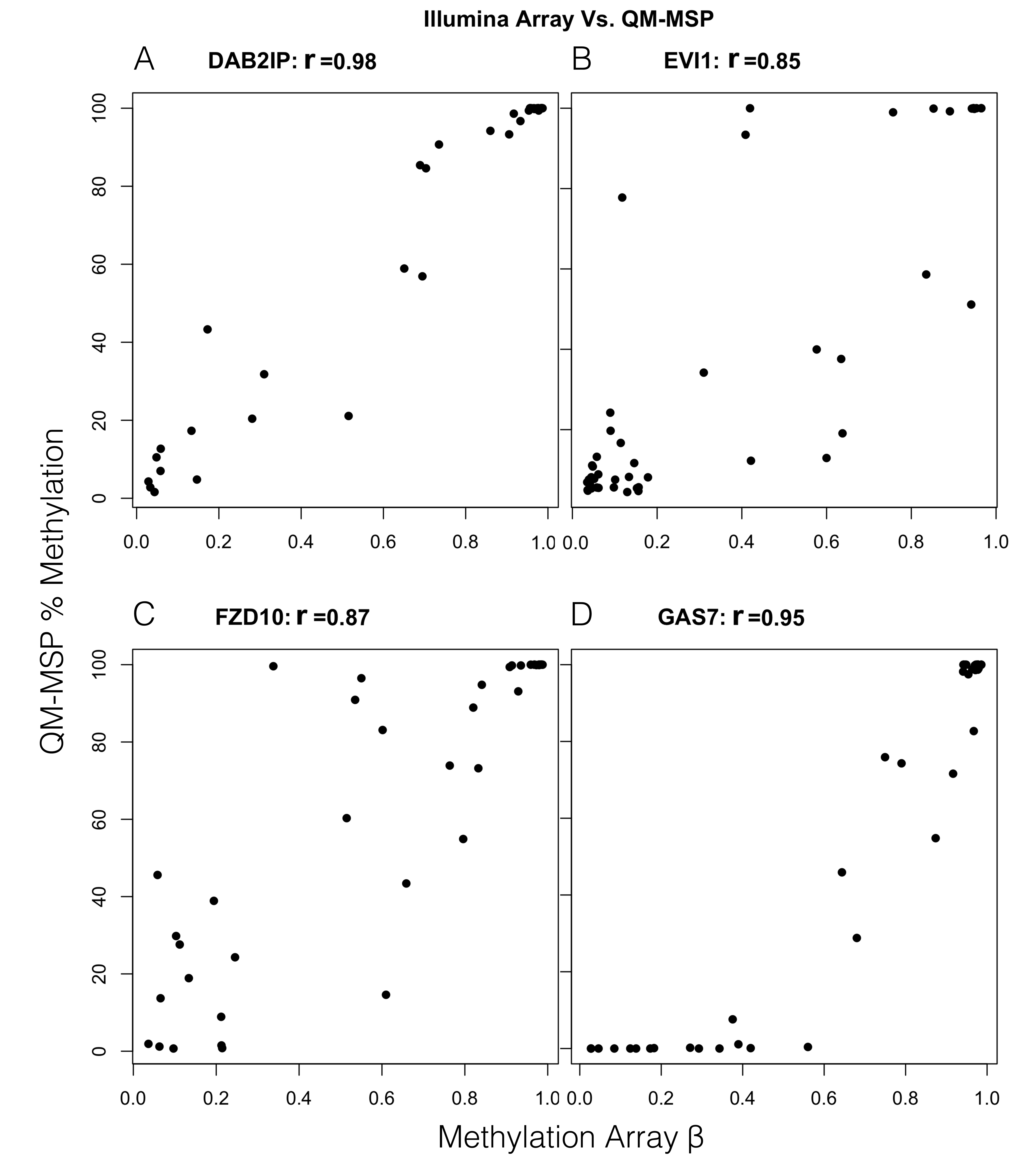

Supplement: Figure S1 — A–D: Validation of array measurements by QM-MSP. Breast cancer cell line methylation levels of selected genes measured by array (x-axis, with β-values ranging from 0–1) and independently by QM-MSP (y-axis, 0–100% methylation). Agreement between assay modalities is measured by Pearson correlation coefficient r. Results for the genes DAB2IP, EVI1, GAS7 and FZD10 are shown in panels A–D, respectively. (TIF) [file pone.0105545.s001.tif]

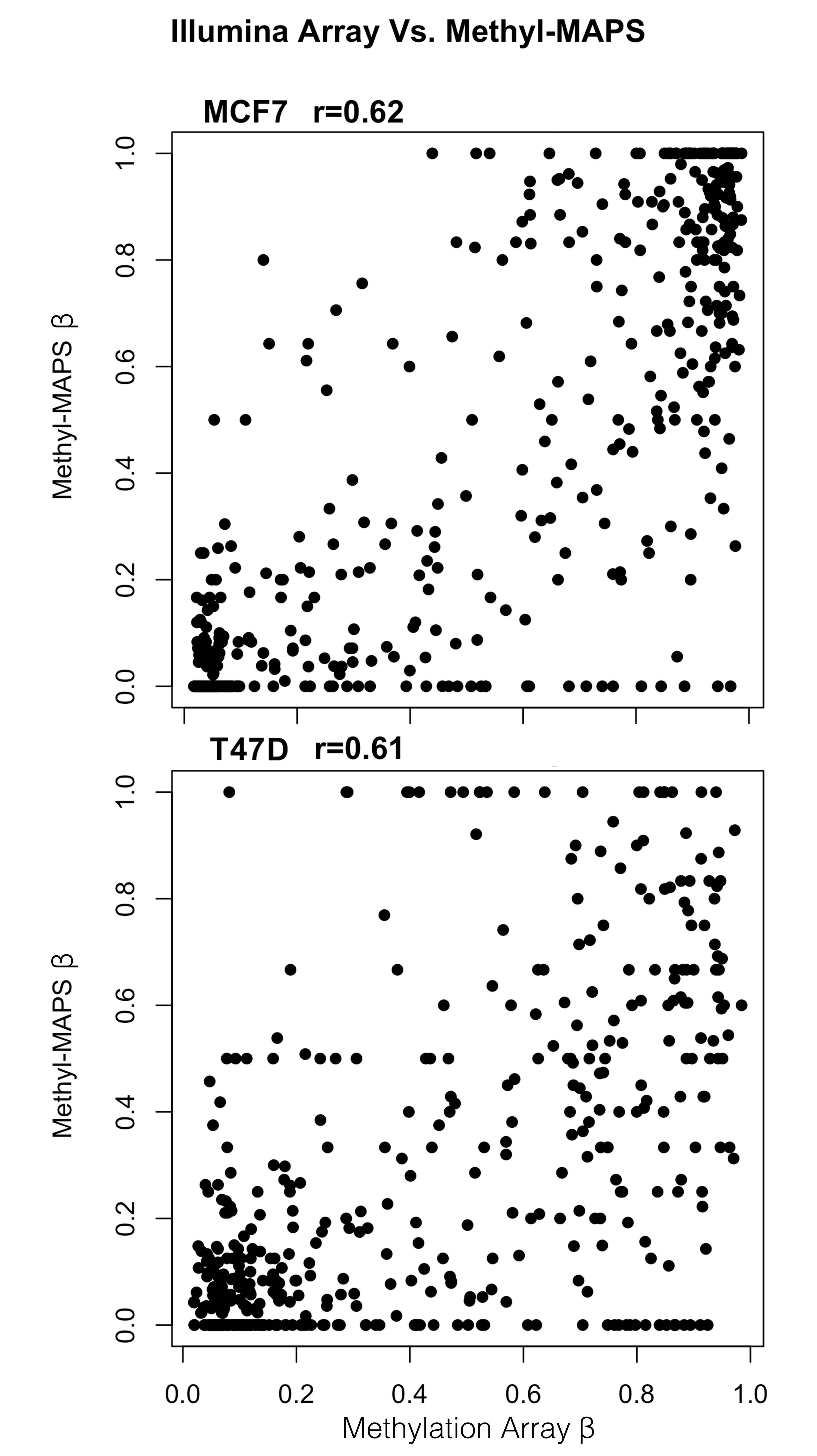

Supplement: Figure S2 — A–B: Validation of array measurements by Methyl-MAPS [22] . Methylation levels of the breast cancer cell lines MCF7 (Panel A) and T47D (Panel B) measured by array (x-axis, with β-values ranging from 0–1) and independently by Methyl-MAPS (y-axis, with β-values ranging from 0–1). CpG sites on the array were selected to be within 100 bp of the corresponding Methyl-MAPS site. Agreement is measured by Pearson correlation coefficient r. (TIF) [file pone.0105545.s002.tif]

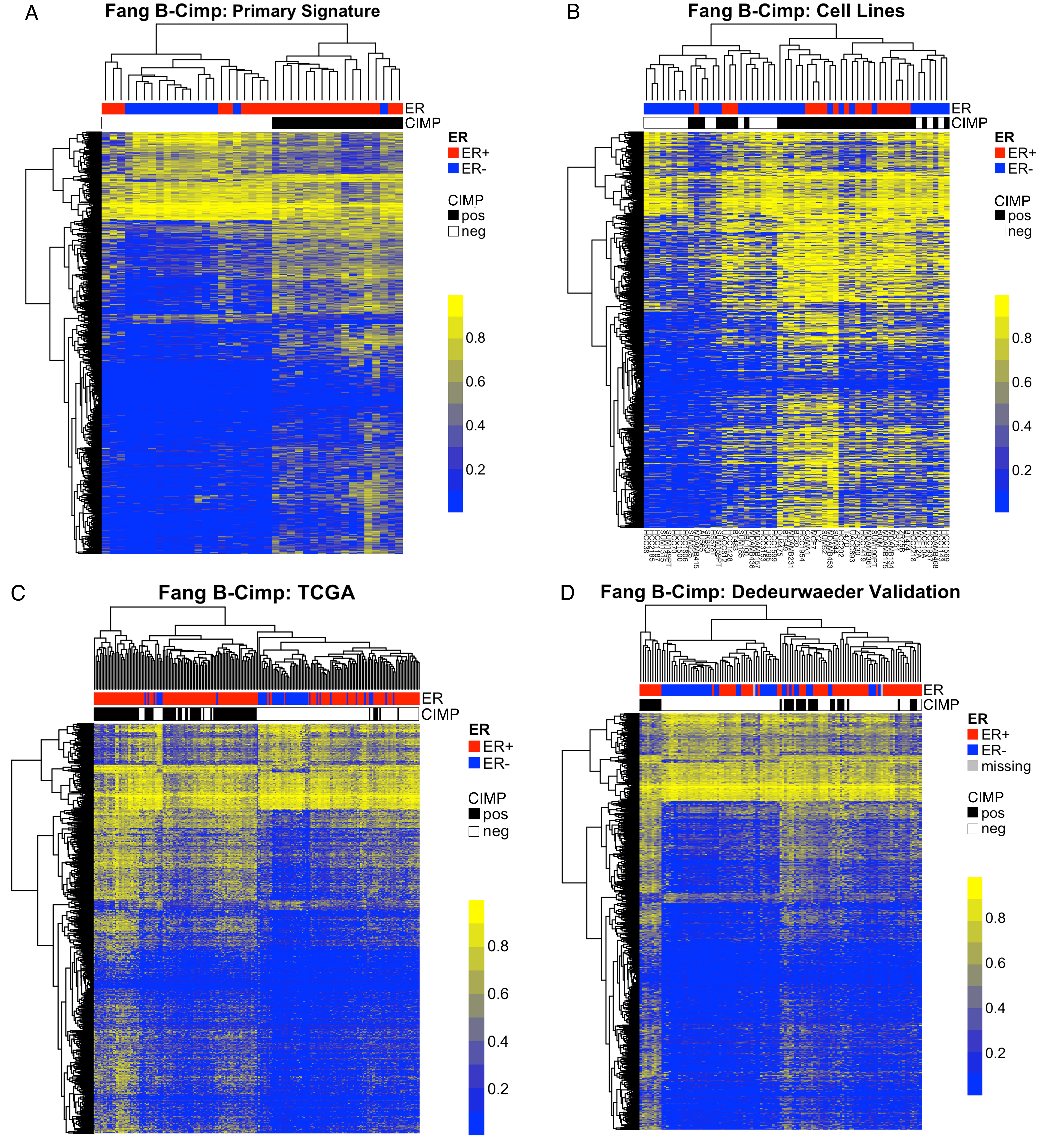

Supplement: Figure S3 — A–D: B-CIMP patterns in primary tumors and cell lines. Panel A shows DNA methylation levels for B-CIMP markers in the Fang tumor samples used to define the B-CIMP signature. ER status, (blue vs red) is indicated along the top margin, along with B-CIMP status (black vs. white). For cell lines (Panel B), the TCGA samples (Panel C), and the Dedeurwaerder validation study (Panel D), CIMP status is inferred as described in Methods and rows in all panels are arranged to match the Fang study in Panel A rather than independently clustered. The methylation level is expressed as a β-value ranging from 0 (no methylation, blue) to 1 (complete methylation, yellow), calculated as described in the Methods section. (TIF) [file pone.0105545.s003.tif]

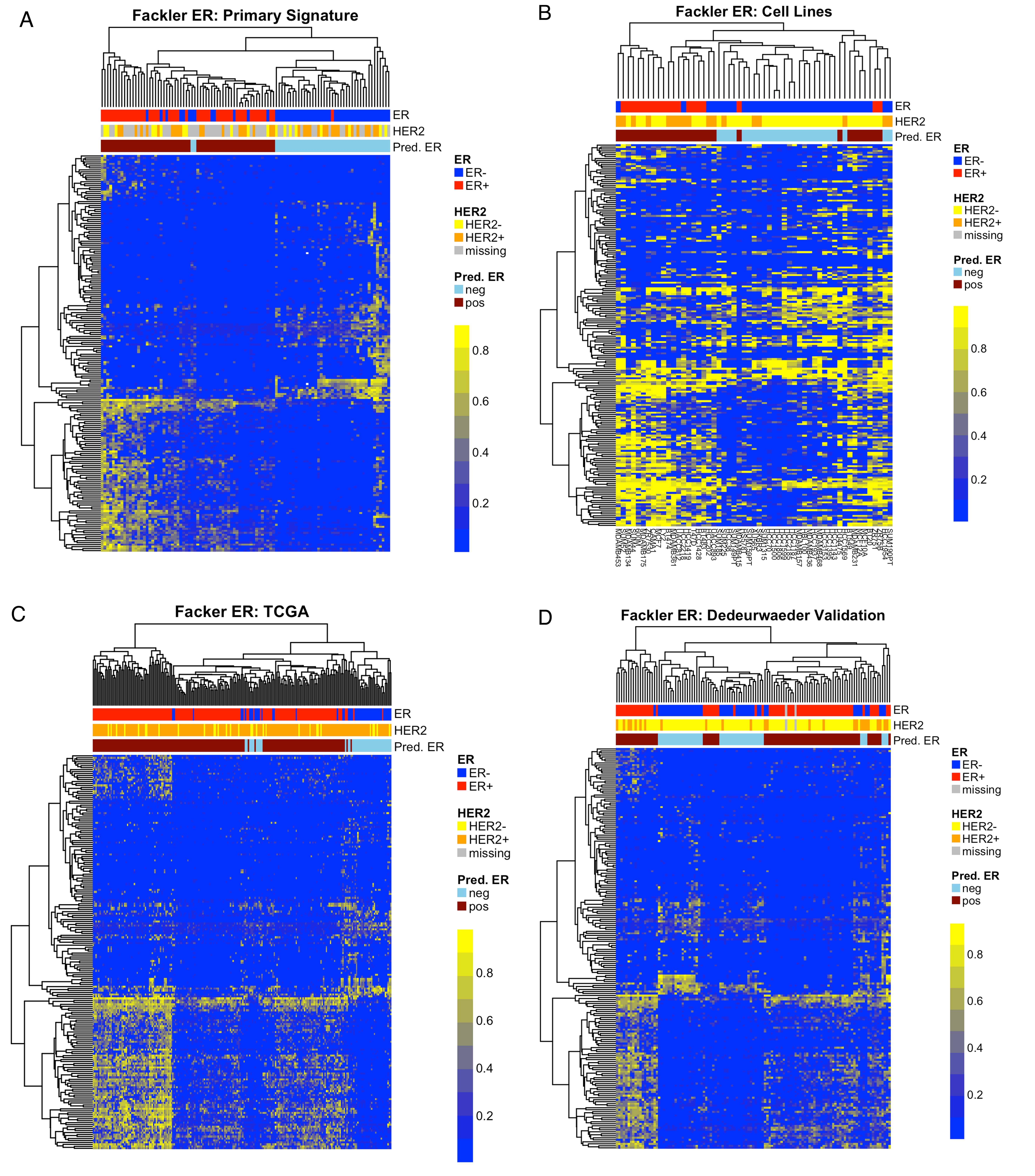

Supplement: Figure S4 — A–D: ER Methylation-signature markers in primary tumors and cell lines. Panel A shows DNA methylation levels for ER markers in the Fackler tumor samples used to define the ER methylation signature [17]. For each tumor, ER status (blue vs red) and HER2 status (yellow vs orange) as defined by immunohistochemistry are indicated along the top margin, along with the ER status predicted by the ER methylation signature as described in methods. For cell lines (Panel B), the TCGA samples (Panel C), and the Dedeurwaerder validation study (Panel D), IHC-defined ER and Her2 status as well as predicted ER status are shown and rows are arranged to match the Fackler study in Panel A rather than independently clustered. (TIF) [file pone.0105545.s004.tif]

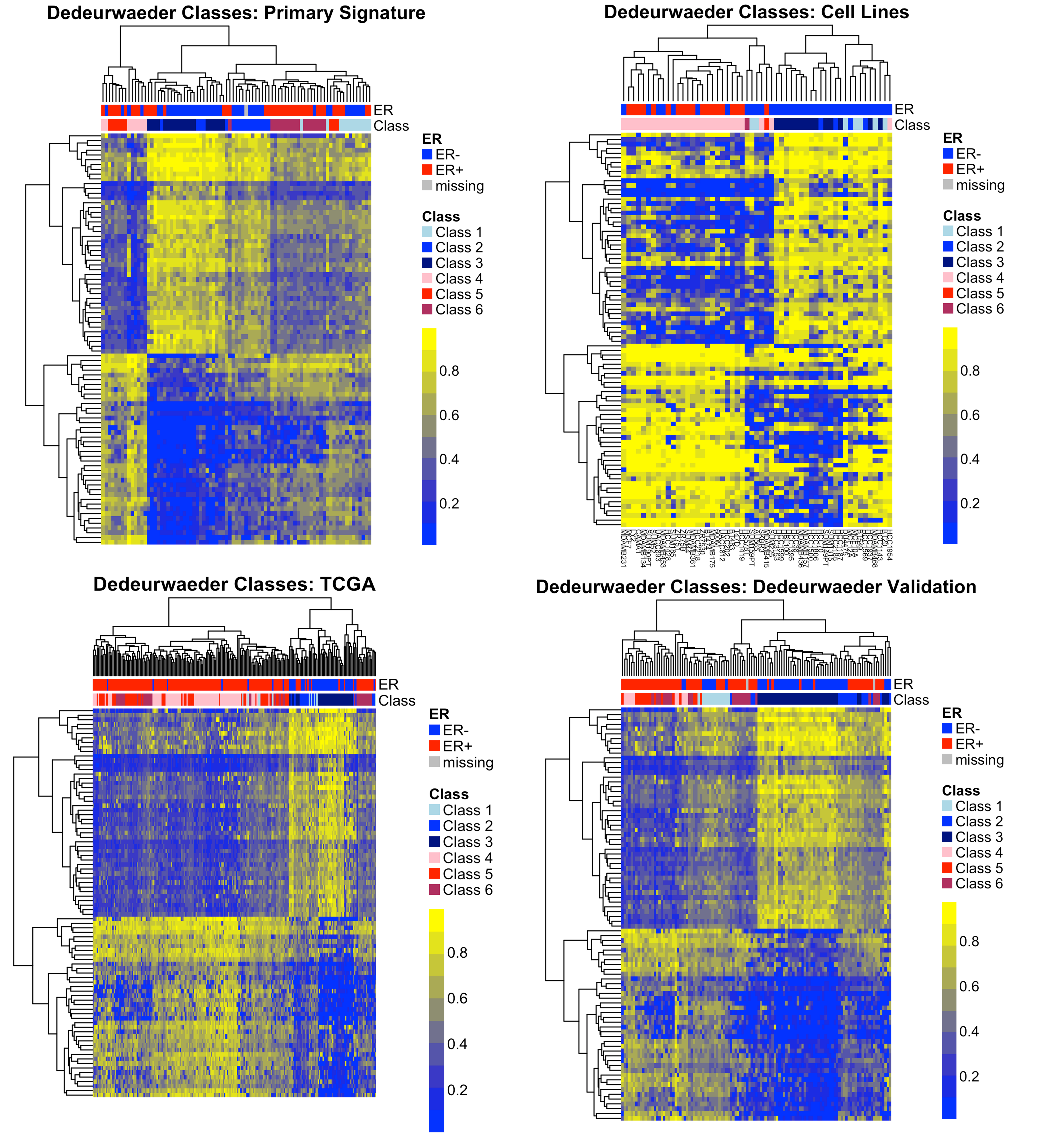

Supplement: Figure S5 — A–D: Markers of methylation-based subtypes in primary tumors and cell lines. Panel A shows DNA methylation levels for markers used to define the signature of the methylation-based subtypes in the Dedeurwaerder tumor samples. ER status as defined by IHC, as well as methylation class, are annotated along the top margin for each tumor sample. For cell lines (Panel B), the TCGA samples (Panel C), and the Dedeurwaerder validation study (Panel D), Dedeurwaerder classes are inferred from the markers as described in methods and rows are arranged to match the Dedeurwaerder study in Panel A rather than independently clustered. (TIF) [file pone.0105545.s005.tif]
